# An Overview of *in vitro* Methods to Study Microglia

**DOI:** 10.3389/fncel.2018.00242

**Published:** 2018-08-06

**Authors:** Raissa Timmerman, Saskia M. Burm, Jeffrey J. Bajramovic

**Affiliations:** ^1^Alternatives Unit, Biomedical Primate Research Centre, Rijswijk, Netherlands; ^2^Genmab B.V., Utrecht, Netherlands

**Keywords:** microglia, cell culture techniques, stem cells, neurodegenerative diseases, *in vitro* models

## Abstract

Neuroinflammation is a common feature in neurodegenerative diseases and strategies to modulate neuroinflammatory processes are increasingly considered as therapeutic options. In such strategies, glia cells rather than neurons represent the cellular targets. Microglia, the resident macrophages of the central nervous system, are principal players in neuroinflammation and detailed cellular biological knowledge of this particular cell type is therefore of pivotal importance. The last decade has shed new light on the origin, characteristics and functions of microglia, underlining the need for specific *in vitro* methodology to study these cells in detail. In this review we provide a comprehensive overview of existing methodology such as cell lines, stem cell-derived microglia and primary dissociated cell cultures, as well as discuss recent developments. As there is no *in vitro* method available yet that recapitulates all hallmarks of adult homeostatic microglia, we also discuss the advantages and limitations of existing models across different species.

## Introduction

Neurodegeneration is defined as the progressive loss of functional neurons. This can be the selective loss of a particular neuronal subtype, such as occurs in diseases as Parkinson’s disease (PD) and amyotrophic lateral sclerosis (ALS), or the widespread loss of many neuronal subtypes, such as occurs in Alzheimer’s disease (AD) and Huntington’s disease (HD). Although all classified as neurodegenerative diseases, the underlying central nervous system (CNS) pathologies are different.

PD pathology is characterized by the formation of Lewy bodies in dopaminergic neurons consisting of fibrillar α-synuclein (Dauer and Przedborski, 2003; Kalia and Lang, 2015), whereas ALS is characterized by protein-rich cytoplasmic inclusions in motor neurons of the spinal cord (Peters et al., 2015; Saberi et al., 2015). AD pathology is characterized by the intracellular accumulation of hyper phosphorylated tau protein and neurofibrillary tangles and by the extracellular deposition of amyloid β (Aβ) in senile plaques (Huang and Mucke, 2012; Castellani and Perry, 2014). HD pathology is characterized by neuronal intranuclear inclusions consisting of mutant huntingtin protein (Ha and Fung, 2012). Although the progress, etiology and symptoms of these diseases differ, neuroinflammation is a common hallmark of all of them.

How neuroinflammation contributes to the progression of neurodegenerative diseases is still unclear as it can either be the cause or the consequence of neuronal cell death. It is, however, generally accepted that persistent inflammation of the CNS is detrimental to neurons. Intriguingly, some molecules that are associated with the pathology of neurodegenerative diseases, such as Aβ and α-synuclein, can induce or modulate inflammatory responses via receptors of the innate immune system (Tahara et al., 2006; Halle et al., 2008; Roodveldt et al., 2010, 2013; Stewart et al., 2010) thereby providing a molecular link between both processes.

Microglia express many receptors of the innate immune system and have a key role in neuroinflammation. Although microglial responses are thought to be primarily neuroprotective, they may also lead to tissue injury and neurodegeneration by the production of pro-inflammatory cytokines and reactive oxygen and nitrogen species (ROS/RNS) (Block et al., 2007; Lijia et al., 2012; Neniskyte and Brown, 2013; Heneka et al., 2014). There is a large body of evidence for microglial activation in the pathogenesis of neurodegenerative disorders (Kim and Joh, 2006; Perry et al., 2010; Crotti and Ransohoff, 2016). Activation of microglia is characterized by an amoeboid morphology, by the production of cytotoxic molecules and pro-inflammatory cytokines, and by the increased expression of complement receptors and histocompatibility complex molecules (Graeber et al., 2011). In the substantia nigra of PD patients, reactive microglia are found along with Lewy bodies (McGeer et al., 1988) and large numbers of activated microglia can be observed in the CNS and spinal cords of human ALS patients as well as in ALS mouse models (McGeer et al., 1993; Hall et al., 1998). Microglia that surround plaques in AD change their morphology from ramified to amoeboid and stain positive for activation markers (Itagaki et al., 1989; Bolmont et al., 2008). Finally, many of the genes that were identified as risk factors for the development of AD in genome-wide association studies such as TREM2, ApoE, ABCA7, PICALM, or CD33 (Karch and Goate, 2015; Crotti and Ransohoff, 2016) are expressed by microglia.

Together, these observations fuel the thought that targeting microglia might provide benefit for those afflicted by neurodegenerative diseases. Detailed cellular biological knowledge of microglia is therefore crucial, and *in vitro* models are instrumental in obtaining such knowledge.

## Microglia Origin, Phenotypes and Functions

Microglia were first described by Rio-Hortega early in the 20th century (Rio-Hortega, 1919) as non-neuronal elements that derive from oligodendroglia and astroglia. Despite intensive research, the origin of microglia has long remained a controversial issue. Researchers described microglia as cells derived from mesodermal pial elements, from pericytes and from neuroectodermal macroglia (Ginhoux and Prinz, 2015). Whereas it was already proposed that microglia derive from yolk-sac macrophages in 1999 (Alliot et al., 1999), conclusive evidence was only provided a decade later when it was shown that microglia originate from yolk-sac primitive myeloid progenitor cells (Ginhoux et al., 2010; Schulz et al., 2012).

In mice, migration and colonization of yolk-sac derived macrophages to and into the brain starts between E8 and E10 (Ginhoux et al., 2010; Schulz et al., 2012). During embryogenesis and throughout adult life, microglia are maintained by local self-renewal without replenishment from hematopoietic progenitors (Ajami et al., 2007; Schulz et al., 2012). Thereby, they form a distinct population from circulating blood monocytes and hematopoietic macrophages (Ajami et al., 2007).

Even under homeostatic conditions, microglia continuously sample the CNS environment with their highly motile processes (Nimmerjahn et al., 2005) and can hardly be described as “resting” but rather as neutral or “M0”. M0 microglia are subject to multiple inhibitory signals from the CNS environment (Butovsky et al., 2014) including that of transforming growth factor beta (TGF-β). TGF-β is a soluble factor with general immunosuppressive properties that is constitutively expressed in the CNS. In addition, cell-cell interactions between microglia and neurons mediated by e.g., CD200R-CD200 contact provide constitutive inhibitory signals to microglia. Loss or disruption of constitutive inhibitory signaling leads to a different microglia phenotype (Hoek et al., 2000; Brionne et al., 2003), which is characterized by the increased expression of activation markers, such as CD11b and CD45. In addition, microglia lose their ramified morphology and can form aggregates, which are normally not observed in healthy CNS tissue (Hoek et al., 2000). This implicates that inhibitory signals from the CNS environment are important to maintain the microglial M0 phenotype. It is important to realize that the M0 phenotype in itself might vary with age and there is also evidence for CNS regional differences (Olah et al., 2011; Holtman et al., 2015; Galatro et al., 2017).

Microglia can switch phenotype when exposed to specific growth factors or cytokines. Classically, *in vitro* exposure to interferon gamma (IFNγ) and/or lipopolysaccharide (LPS) has been associated with morphological alterations from ramified to amoeboid and with the induction of an activated or “M1” phenotype. This phenotype has long been associated with neuroinflammation. Alternatively, *in vitro* exposure of microglia to anti-inflammatory cytokines like interleukin (IL)-4 can induce an alternative, “M2” phenotype. More recent studies have demonstrated that macrophages and microglia can display a wide spectrum of intermediate phenotypes, both *in vitro* as well as *in vivo* (Olah et al., 2011; Vogel et al., 2013; Murray et al., 2014; Peferoen et al., 2015). The recent identification of the ApoE pathway as a driver of microglial phenotype alteration in AD (Krasemann et al., 2017) has casted further doubt on the usefulness of the classical dichotomous M1-M2 phenotype description (Ransohoff, 2016).

As resident innate immune cells of the brain, microglia provide the first line of defense against invading pathogens, such as viruses, bacteria and prions (Rock et al., 2004; Ousman and Kubes, 2012). Like other macrophages, microglia are phagocytic cells that can secrete a wide range of chemokines and pro- and anti-inflammatory cytokines (Kettenmann et al., 2011). While microglial inflammatory responses have been the focus of much research, there is increasing appreciation of the contribution of microglia to CNS development and homeostasis. Microglia secrete neurotrophic and growth factors that regulate the proliferation of oligodendrocytes, astrocytes and neuronal progenitors, and they contribute to the maturation of neural circuits (Schafer and Stevens, 2015). During CNS development and adult neurogenesis, microglia contribute to the clearance of superfluous neurons (Neumann et al., 2009; Marin-Teva et al., 2011; Cunningham et al., 2013). Furthermore, microglia are involved in synapse elimination or synaptic pruning, which is required to establish efficient neuronal networks (Paolicelli et al., 2011). Inappropriate synaptic connections are tagged by complement components C1q and C3 (Stevens et al., 2007), and can be recognized by microglia that express the complement C3 receptor (Schafer et al., 2012).

## *In Vitro* Microglia Models

As most neurodegenerative diseases develop at adult age, *in vitro* models that aim to study the role of microglia in the pathogenesis of neurodegenerative disorders should ideally recapitulate the M0 phenotype of adult or elderly human microglia. This will allow exposure to different pathology-associated stimuli in order to characterize microglial responses and to test therapeutic interventions in such responses. Recent publications of RNA transcriptome profiles of *ex vivo* M0 microglia from different species and age (Butovsky et al., 2014; Zhang et al., 2014; Galatro et al., 2017; Gosselin et al., 2017; Olah et al., 2018) have opened up previously shut doors. Not only have these studies led to the identification of microglia-specific markers such as P2RY12 and TMEM119, they have also provided a blueprint of the RNA transcriptome profile that *in vitro* microglia should ideally express.

Over the years various microglia *in vitro* models have been developed, including microglia cell lines, stem cell-derived microglia cultures and primary dissociated cell cultures. Each of these models has specific advantages and limitations, which will be discussed in detail.

### Microglia Cell Lines

Microglia cell lines are available from mouse, rat, macaque and human origin (**Table 1**). Most of these lines stem from primary microglia cultures derived of the brain or the spinal cord, which were immortalized by viral transduction with oncogenes (e.g., v-myc, v-raf, v-mil, SV40 T antigen). Non-transformed microglia cell lines that stem from primary microglia precursor cell cultures have been described as well. Advantages of cell lines include their ease of maintenance and their abundant availability due to their unrestricted proliferative capacity. A major disadvantage is their susceptibility to dedifferentiation. Furthermore, viral transformation or immortalization may alter the microglial phenotype. Recent studies have pointed out that microglia cell lines differ both genetically and functionally from primary microglia and *ex vivo* microglia (Butovsky et al., 2014; Das et al., 2016; Melief et al., 2016). In addition, microglial cell lines obtained from neonatal or embryonic CNS sources are unlikely to reflect the phenotype of adult or elderly microglia. Despite these limitations, microglia cell lines are suitable for e.g., biochemical and molecular approaches as well as for high-throughput screening assays which all require high cell numbers.

**Table 1:** Overview of available microglia cell lines of mouse, rat, rhesus macaque and human origin.

Species	Cell line	Donor age	Brain area	Immortalization procedure	Citations per 2018	Reference
Mouse	BV2	Neonatal	Cerebral cortex	Transformed, *v-raf/v-myc* oncogene	>750	Blasi et al., 1990
	C8–B4	Neonatal	Cerebellum	Spontaneous	15	Alliot et al., 1996
	EOC-2, EOC-13.31, EOC-20	Neonatal	Whole brain	Spontaneous, M-CSF-dependent clones	29	Walker et al., 1995
	IMG	Adult	Whole brain	Transformed, *v-raf/v-myc* oncogene	2	McCarthy et al., 2016
	MG5	Neonatal	Cerebral cortex	Transformed, microglia derived from p53-deficient mice	14	Ohsawa et al., 1997
	MG6	Neonatal	Whole brain	Transformed, *c-myc* oncogene	14	Takenouchi et al., 2005
	MG20	Neonatal	Whole brain	Transformed, *c-myc* oncogene	3	Iwamaru et al., 2007
	Muμglia	Adult	Cortex	Transformed, SV40 large T antigen (and hTERT)	1	Garcia-Mesa et al., 2017
	N3, N9, N11, N13	Embryonic	Whole brain	Transformed, *v-myc* or *v-mil* oncogenes, clones	>200	Righi et al., 1989
	RA2	Neonatal	Whole brain	Non-enzymatic and non-virus transformed, GM-CSF-dependent	14	Sawada et al., 1998
	SIM-A9	Neonatal	Cerebral cortex	Spontaneous	4	Nagamoto-Combs et al., 2014

Rat	HAPI	Neonatal	Cerebral cortex	Spontaneous	45	Cheepsunthorn et al., 2001
	MLS-9	Neonatal	Neocortex	Spontaneous	17	Zhou et al., 1998

Macaque	Mqμglia	Adult	Cerebral cortex	Transformed, SV40 large T antigen (and hTERT)	1	Garcia-Mesa et al., 2017

Human	*CHME-5*	*Embryonic*	*Spinal cord/cortex*	*Transformed, transfection with SV40 large T antigen*	25	Janabi et al., 1995
	HMO6	Embryonic	Telencephalon	Transformed, *v-myc* oncogene	11	Nagai et al., 2001
	Huμglia	Adult	Cortex	Transformed, SV40 large T antigen (and hTERT)	1	Garcia-Mesa et al., 2017

*In italics the CHME-5 cell line, of which the exact origin is currently uncertain.*

#### Mouse Cell Lines

A large variety of mouse microglia cell lines has been generated over the years. We provide a non-exhaustive list in **Table 1** and will briefly discuss the two most used mouse cell lines, BV2 and N9. The BV2 cell line was generated by transduction of neonatal primary microglia with the v-raf/v-myc carrying J2 retrovirus. Immortalized, transformed cells survived for more than 4 weeks in culture, while non-transformed cells died (Blasi et al., 1990). BV2 cells express macrophage markers such as macrophage antigen (MAC) 1 and MAC2, and are negative for the astrocyte marker glial fibrillary acidic protein (GFAP) and the oligodendrocyte marker galactocerebroside (GalC). The expression of MAC2 (also known as galectin-3) in particular is important when considering murine microglial cell lines to model neuroinflammatory processes (Burguillos et al., 2015; Yip et al., 2017). BV2 cells have been used for many years to study neuroinflammation and neurodegenerative disorders, including AD and PD (Stansley et al., 2012; Gao et al., 2013; Griciuc et al., 2013). BV2 cells are responsive to LPS, they have phagocytic capabilities and increase their expression levels of ROS/RNS and pro-inflammatory genes after exposure to Aβ fibrils or to α-synuclein (Stansley et al., 2012; Boza-Serrano et al., 2014).

Embryonic primary microglial cultures that were transformed with v-myc or v-mil oncogenes of the avian MH2 retrovirus led to the generation of a series of clonally derived microglial cell lines, of which N9 is the most well studied (Righi et al., 1989). N9 cells express Immunoglobulin G receptors, glycoprotein F4/80 and MAC1, and are negative for GFAP and GalC. Similar to BV2 cells, N9 cells produce and secrete pro-inflammatory cytokines after LPS stimulation and can phagocytose Aβ fibrils (Stansley et al., 2012).

#### Rat Cell Lines

The highly aggressively proliferating immortalized (HAPI) cell line is derived from neonatal primary cultures enriched for microglia and was the first cell line recognized to be the result of spontaneous immortalization (Cheepsunthorn et al., 2001). The genetic mutation that is responsible for immortalization is unknown yet. HAPI cells express the microglial markers isolectin B4, OX-42 and fructose transporter GLUT5, and are negative for GFAP and the oligodendrocyte marker A2B5 (Cheepsunthorn et al., 2001). Exposure to LPS induces secretion of tumor necrosis factor α and production of ROS/RNS, and HAPI cells are capable of phagocytosis as demonstrated by the uptake of fluorospheres that were added to the culture medium (Cheepsunthorn et al., 2001).

Cell lines derived from rodents are frequently being used to study microglial functions. As recent studies have revealed important differences between rodent microglia cell lines and primary microglia (Horvath et al., 2008) as well as between rodent and human microglia both in terms of aging and function (Smith and Dragunow, 2014; Galatro et al., 2017), results obtained with rodent microglia cell lines should always be extrapolated with care.

#### Human Cell Lines

The HMO6 cell line is a human microglial cell line generated by transduction of embryonic primary microglia from telencephalon tissue with a v-myc carrying PASK 1.2 retroviral vector. Of the transducted cells, 99% remained positive for the macrophage/microglia markers *Ricinus communis* agglutinin-1 lectin and CD11b, and negative for the neuronal marker neurofilament-medium, the astrocyte marker GFAP and the oligodendrocyte marker myelin basic protein (Nagai et al., 2001). ATP responsiveness and phagocytotic capacity of HMO6 cells were grossly comparable to that of human embryonic primary microglia. However, detailed characterization of the induced protein profile after exposure to LPS or Aβ_25-35_ revealed that HMO6 cells secreted a markedly less diverse cocktail of soluble mediators compared to primary microglia (Nagai et al., 2001). Although gene expression analysis demonstrated that after 6 h exposure to LPS or Aβ_25-35_, mRNA expression levels of most soluble inflammatory mediators were enhanced in HMO6 cells comparable to levels in primary microglia, exposure to LPS did not induce the expression of mRNA encoding for macrophage inflammatory protein (MIP)-1α or introduce tumor necrosis factor alpha (TNF-α) in HMO6 cells whereas it did in primary microglia (Nagai et al., 2001). More importantly, exposure of primary human microglia to LPS induced the secretion of IL-1β, IL-6, IL-8, TNF-α, and MIP-1α proteins, and exposure to Aβ_25-35_ induced the secretion of IL-1β, IL-8, TNF-α, and MIP-1α proteins. Exposure of HMO6 cells to LPS or Aβ_25-35_ only triggered the secretion of IL-8 and TNF-α proteins (Nagai et al., 2001). Whether this lack of responsiveness is attributable to the transformation process is unclear yet. Although the HMO6 cell line has long been the only human microglia cell line available, the patented status of the cells has most probably inhibited its widespread use.

Other immortalized human microglial cell lines that have been described include HMC3 (Janabi et al., 1998) and C13NJ (Martin et al., 2003), which both originate from the CHME-5 cell line (Janabi et al., 1995). Importantly, a recent study has found evidence that CHME-5 cells are not of human, but of rat origin (Garcia-Mesa et al., 2017), implying that also the HMC3 and C13NJ cell lines are of rat origin. This warrants further investigation and until this is elucidated these cell lines should be used with caution. Interestingly, the group that uncovered the origin of CHME-5 has itself developed a method to immortalize adult primary microglia from mouse, macaque and human origin (Garcia-Mesa et al., 2017), transforming the cells with either the SV40 large T antigen alone or in combination with human telomerase reverse transcriptase (hTERT).

### Stem Cell-Derived Microglia

Stem cell technology not only holds great promise for regenerative medicine, it can also provide scientists with an unlimited availability of cells for *in vitro* purposes. The two types of stem cells most often described in the context of microglia are embryonic stem cells (ESCs) and induced pluripotent stem cells (iPSCs). ESCs are derived from the inner cell mass of a blastocyst, whereas iPSCs can be generated from adult cells (e.g., fibroblasts) by reprogramming them via overexpression of just four transcription factors (Takahashi and Yamanaka, 2006). A major advantage of the iPSC approach is that it allows comparisons of iPSC-derived cells from healthy donors and patients with neurological disorders. Thereby, the genetic background of these patients is recapitulated in their iPSC-derived neurons and glial cells.

Many protocols have been established to differentiate ESCs and iPSCs to specific neuronal lineages, such as neurons, astrocytes and oligodendrocytes (Nistor et al., 2005; Krencik and Zhang, 2011; Zhang et al., 2013). Microglia have, however, proven to be amongst the most difficult cells to differentiate from stem cells, partly because their exact origin remained obscure until 2010. The first microglia-related stem cell studies described methods to differentiate mouse ESCs to microglia by directing ESCs through neuronal differentiation pathways (Tsuchiya et al., 2005; Beutner et al., 2010). Since lineage tracing studies in mice revealed that microglia originate from primitive yolk-sac macrophages (Ginhoux et al., 2010), more recent protocols direct differentiation of ESCs and iPSCs to embryonic macrophage precursors first, before skewing these toward a microglial phenotype.

**FIGURE 1:**
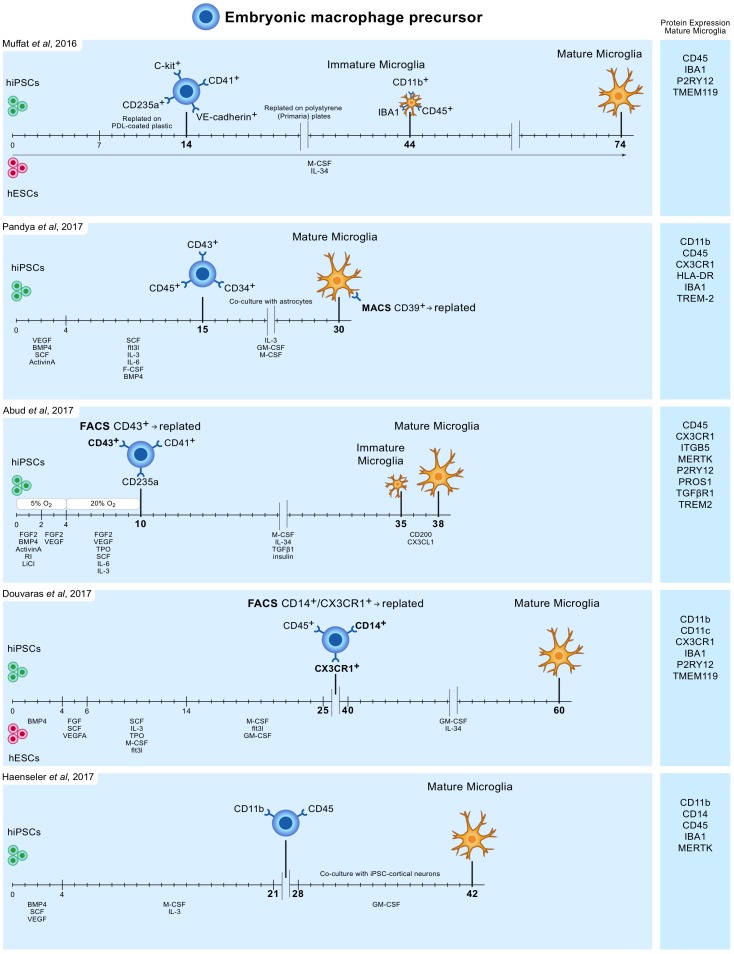
Overview of protocols to differentiate human iPSC and ESC to microglia-like cells. Timelines are indicated in days. hiPSC and hESC are first differentiated to embryonic macrophage precursor cells, which is consistent with the *in vivo* developmental lineage of microglia. Subsequently, these precursor cells are differentiated into mature microglia-like cells.

In 2016 Muffat and colleagues described a method (Muffat et al., 2016) in which they differentiated human ESCs and iPSCs into neuralized embryonic bodies (EB) and cystic EBs. When plated on poly-D-lysine for 14 days, the cystic EBs became positive for markers of early yolk-sac myelogenesis, such as PU.1. Yolk-sac EBs were collected and replated in polystyrene plates for 30 days after which semi-adherent round cells appeared that were highly phagocytic and highly motile. Subsequently, these cells were kept in culture for 30 days to mature into microglia-like cells, bringing the total culture time to around 75 days (**Figure 1**). Transcriptome analysis showed that the mRNA expression profile resembles that of human fetal microglia.

Pandya and colleagues reported the differentiation of human iPSCs into microglia-like cells in around 30 days by exposure to defined factors followed by co-culture with astrocytes (Pandya et al., 2017). First, iPSCs were cultured for 4 days in medium containing specific VEGF, BMP4, SCF, and ActivinA, after which at day 4, 7, and 10, cells were exposed to another type of defined medium. On day 15, cells expressed CD34, CD45, and CD43, which are markers for myeloid progenitor cells. These cells were further co-cultured with human astrocytes in medium containing IL-3, granulocyte-macrophage colony-stimulating factor (GM-CSF) and macrophage colony-stimulating factor (M-CSF). After another 1 to 2 weeks of culture, these cells differentiated into microglia-like cells (**Figure 1**). Transcriptome analysis demonstrated that the gene expression profile of these iPSC-derived microglia resembles that of human fetal microglia, but also that of dendritic cells and macrophages. This immature phenotype warrants caution when considering the use of these cells to model adult microglia in neurodegenerative diseases.

Around the same time Abud and colleagues published a protocol describing how iPSCs can be differentiated into microglia-like cells in just over 5 weeks (Abud et al., 2017). First, iPSCs were differentiated into CD43^+^ myeloid progenitors for 10 days using defined medium and temporal exposure to low (5%) oxygen levels (a procedure often used for the generation of myeloid progenitor cells). After 10 days, medium was changed to serum-free microglia differentiation media containing M-CSF, IL-34, TGF-β1, and insulin. At day 35, microglia-like cells were exposed to CD200 and CX3CL1 for 3 days to induce maturation (**Figure 1**). Gene expression analysis demonstrate that these microglia-like cells cluster with human fetal and adult primary microglia.

Douvaras and colleagues published a protocol in which ESCs and iPSCs were first differentiated into myeloid progenitor cells by serial exposure to defined media (Douvaras et al., 2017). Myeloid progenitor cells were purified, replated and exposed for 2 weeks to IL-34 and GM-CSF, which resulted in differentiation into microglia-like cells after around 60 days of total culture (**Figure 1**). The obtained ESC/iPSC-derived microglia express microglia markers and resemble human fetal primary microglia by gene expression profile.

The simplest protocol published describes a period of around 6–8 weeks to generate, with high yields, microglia from iPSCs (Haenseler et al., 2017). The protocol is relatively simple and avoids repeated replating or cell sorting. iPSCs were grown into EBs, and after approximately a month embryonic macrophage precursors emerged in the culture supernatants. These cells were harvested and subsequently co-cultured for 2 weeks with iPSC-derived cortical neurons. The obtained microglia-like cells are phagocytic, adopt a highly dynamic ramified microglia-like morphology and have a transcriptional profile similar to that of human fetal primary microglia. On the neuronal side, co-culture with iPSC-derived macrophage precursors retained neuronal maturity and functionality for at least 42 days (Haenseler et al., 2017).

As this is a new, rapidly emerging field, there is no consensus on methodology to generate iPSC or ESC-derived microglia yet. A variety of different culture media and culture conditions have been used, and comparative studies and harmonization are necessary to further validate the most reliable and reproducible approaches. Although stem cell technology can provide researchers with a readily available source of microglia, it should also be taken into account that these cells have never been exposed to the CNS microenvironment. How this lack of exposure to CNS-specific environmental cues might affect microglial differentiation and function will be discussed below. In addition, most neurodegenerative disorders develop at adult or elderly age and it is therefore important to recapitulate age-related characteristics in microglia when neurodegenerative disorders are studied. However, reprogrammed iPSCs from adult donors have had their aging signature, such as telomere attrition and cellular senescence, reset. Direct reprogramming of somatic cells to microglia might tackle this problem by avoiding passage through the stem cell phase. It has been demonstrated that direct reprogramming retains aging-associated transcriptomic signatures (Mertens et al., 2015; Prasad et al., 2016).

### Primary Microglia

Methods to generate dissociated single cell cultures of primary microglia have been described for mice, rats, non-human primates and humans (**Table 2**). Most methods start with mechanical and enzymatic dissociation of the donor brain tissue followed by a density gradient centrifugation step to separate the myelin from the cells. Dependent on the density gradient used, this can either be sufficient to obtain microglia cultures with a purity of > 99% or it is followed by additional purification steps (Cardona et al., 2006; Zuiderwijk-Sick et al., 2007). Other purification steps used to isolate microglia include labeling of cells with antibody-coated magnetic beads followed by magnetic-activated cell sorting (MACS) (Nikodemova and Watters, 2012; Mizee et al., 2017), labeling of cells with fluorescently labeled antibodies followed by fluorescence-activated cell sorting (FACS) (Olah et al., 2012; Bennett et al., 2016) or specific shaking procedures (Tamashiro et al., 2012). Primary microglia from mice and rats are generally derived from brain tissue of neonatal animals (Giulian and Baker, 1986), although more studies are now reporting the use of adult animals as well (Butovsky et al., 2014). The advantage of using rodent primary microglia is that these animals form a genetic homogenous, specific pathogen free (SPF) population where *ante-mortem* conditions and *post-mortem* delay can be tightly controlled. The use of primary microglia derived from transgenic mice has been instrumental in delineating the role of specific genes in microglia activation. Limitations of rodent primary microglia include their evolutionary divergence from humans and lack of heterozygosity due to inbreeding and their aseptic housing conditions (Smith and Dragunow, 2014). Differences between rodents and humans have been described to hamper translation of rodent (neuro) immunological studies to the clinic (Bajramovic, 2011; Seok et al., 2013; Smith and Dragunow, 2014).

**Table 2:** Comparison of human, non-human primate, and rodent primary microglia cell culture features.

	Human	Non-human primate	Rodent
Genetic distance to humans	None	Close evolutionary proximity to humans	Considerable evolutionary divergence from humans
Breeding	Outbred	Outbred	Inbred
Environment	Non-SPF	Non-SPF	SPF
Ante mortem conditions	Uncontrollable and often unknown	Controllable and well described	Controllable and well described
Post-mortem delay	4–24 h at best	None	None
Donor age (most often)	Fetal or aged adults	Adult	Fetal/neonatal
Donor characteristics (most often)	Neurological disease, shortage of non-diseased donors	Free of neurological diseases	Free of neurological diseases
Availability	Limited: brain banks	Limited: primate centers	Widely available
Microglia yields	0.1–0.5^∗^10^6^ cells/gram wet brain tissue; often 1-2 g available (Olah et al., 2012)	0.6–1.2^∗^10^6^ cells/gram wet brain tissue; 25 g available (Zuiderwijk-Sick et al., 2007)	0.3–1^∗^10^6^ cells/brain; can be pooled from multiple brains of inbred animals (Ni and Aschner, 2010)
Availability other tissues from the same donor	Limited	Good	Good

Dissociated cultures of human primary microglia can either be derived from fetal tissue that becomes available after abortion or from *post-mortem* brain tissue that becomes available from deceased human donors (Durafourt et al., 2013; Melief et al., 2016; Rustenhoven et al., 2016; Mizee et al., 2018). Isolation from brain material of patients who suffered neurological disease may provide new insights into the role of microglia in the pathogenesis (Perlmutter et al., 1992; Kim and Joh, 2006; Rogers et al., 2007; Peferoen et al., 2015). To enable research using human brain tissue, brain banks have been set up worldwide. Human primary microglia are derived from different individuals reflecting the genetic variability within a population and translation of results is not hampered by the use of a genetically divergent species (Smith and Dragunow, 2014). Limitations of human primary microglia include the limited availability of (healthy) human brain tissue, and the limited control over the *ante mortem* conditions and *post-mortem* delay (Watkins and Hutchinson, 2014), which might affect the microglia phenotype. For example, CD11b expression, a marker for immunoreactivity, shows a significant positive correlation with post-mortem delay in gray matter microglia (Mizee et al., 2017).

To bridge the gap between rodents and humans, primary microglia cultures derived from non-human primates may be considered. Protocols have been developed for the rhesus macaque (*Macacca mulatta)* (Zuiderwijk-Sick et al., 2007). To isolate such cells, the presence of a research center with non-human primate facilities is a requirement, which might be considered as a limitation. Advantages on the other hand are that microglia are isolated from outbred individuals that are in close evolutionary proximity to humans with much control over *ante mortem* conditions and *post-mortem* delay. Comparison of primary microglia with primary bone marrow-derived macrophages from the same donors has been instrumental in uncovering microglia specific features of innate immune responses (Van Der Putten et al., 2012; Burm et al., 2015, 2016), demonstrating the utility of this methodology.

## Culture Conditions and the CNS Microenvironment

At least equally important as the method of isolation, are the *in vitro* culture conditions. Both for stem cell-derived microglia as well as of primary dissociated microglia, many different cell culture media combined with a diversity of growth factors have been tested. Data from knock out mouse studies demonstrated the importance of the colony stimulating factor-1 (CSF-1) receptor for microglial survival and proliferation (Erblich et al., 2011) leading to the inclusion of CSF-1 (M-CSF) in most microglia media. The discovery of IL-34 as a second, brain-specific, ligand for the CSF-1 receptor has inspired researchers to experiment with this factor as well (Lin et al., 2008). In addition, RNA transcriptome comparisons of *ex vivo* microglia with *in vitro* microglia identified the TGF-β pathway as important (Butovsky et al., 2014; Bohlen et al., 2017). These data also demonstrated that the RNA transcript profiles of *ex vivo* microglia differed considerably from those of *in vitro* microglia. A recent publication describes that at least part of this difference can be attributed to culturing in the presence of serum (Bohlen et al., 2017). Besides the fact that *in vivo*, in a healthy CNS, microglia are not exposed to serum, serum exposure has more disadvantages. Serum is a poorly defined cell culture component and batch-to-batch variability negatively contributes to reproducibility. *In vitro* cultures of adult primary microglia under serum-free conditions are, however, characterized by low proliferative capacity and decreased survival rates (Bohlen et al., 2017). Identification of cholesterol as the minimal component to confer increased survival of microglia in the absence of serum is a major step forward toward better defined cell culture conditions (Bohlen et al., 2017). The RNA transcript profile of *ex vivo* M0 microglia will continue to provide us with clues on cell culture conditions to better mimic this profile *in vitro*, and it is reasonable to expect major modifications to both stem-cell derived as well as primary microglial cell culture conditions in the near future.

Research on the importance of the CNS microenvironment in retaining the M0 phenotype of microglia has gained momentum over the past years. Regretfully, microglial cell lines have long lost their specific microenvironmental input and stem cell-derived microglia have never even received it. But also adult primary microglia, although they have been “educated” in the CNS microenvironment for the life span of the donor, are deprived of microenvironmental cues the moment they are brought in culture. Interestingly, loss of the CNS-specific signature is at least partly reversible by engraftment of isolated microglia into a CNS parenchyma that lacks microglia (Masuch et al., 2016). Fascinatingly, also bone marrow-derived macrophages acquire microglia-like features when they are confronted with a CNS parenchyma (Hinze and Stolzing, 2011). Together these findings demonstrate that signals from the CNS microenvironment are required to sustain microglial specification (Bohlen et al., 2017). High-throughput technologies for studying the transcriptome, epigenome and proteome have been used to study the effect of the local environment on microglial responses (Butovsky et al., 2014; Gosselin et al., 2014, 2017; Amit et al., 2015; Bohlen et al., 2017) confirming that loss or disruption of homeostatic CNS microenvironment signals affects the microglia phenotype (Hoek et al., 2000; Butovsky et al., 2014; Bohlen et al., 2017).

Many of these inhibitory signals are provided by cell-cell interactions between microglia and neurons, astrocytes and the oligodendrocyte/myelin complex (Bajramovic, 2011). CD200 e.g., is expressed by astrocytes and neurons and interacts with the CD200 receptor that is expressed by microglia. Ligand binding suppresses LPS-induced cytokine and IFN production by inhibition of extracellular signal-regulated kinase, mitogen-activated protein kinase p. 38 and c-jun *N*-terminal kinase (Jenmalm et al., 2006). If this constitutive inhibitory signal is lost, microglia acquire an activated phenotype (Hoek et al., 2000; Brionne et al., 2003). Another example is CD172a (also known as SIRPα), an inhibitory receptor expressed by microglia. Its ligand CD47 is abundantly expressed in the brain, and CD172a-CD47 interactions provide constitutive suppressive signals to microglia (Kharitonenkov et al., 1997; Kierdorf and Prinz, 2013). Finally, the triggering receptor on myeloid cells 2 (TREM2) that is expressed on microglia plays a role in inhibition of Toll-like receptor (TLR) mediated signaling. Loss or disruption of TREM2 expression results in increased secretion of pro-inflammatory cytokines in response to various TLR ligands (Hamerman et al., 2006). Interestingly, loss-of-function mutations of TREM2 cause the rare genetic disorder Nasu-Hakola disease (NHD) (Xing et al., 2015). NHD is a leukodystrophy characterized by progressive presenile dementia. Pathological examination of the CNS of NHD patients shows atrophy of the cerebral white matter and sclerotic lesions that contain activated microglia (Paloneva et al., 2001).

It is a major challenge to provide dissociated microglia with the mix of environmental cues that they receive *in vivo.* Culturing microglia in the presence of astrocyte-conditioned medium or on an astrocyte feeder layer supports microglia survival and the development of a ramified phenotype (Tanaka and Maeda, 1996; Bohlen et al., 2017). An alternative approach is to use 3D cell culture models in which different CNS cells are included (Hopkins et al., 2015; Schwartz et al., 2015; Watson et al., 2017). For example, brain organoids can be used as a supportive environment. Abud and colleagues elegantly demonstrated that iPSC-derived microglia naturally integrate into the 3D structure of brain organoids. After integration in the organoid, microglia were able to mature, ramify, and to respond to injury similar to *in vivo* microglia (Abud et al., 2017). In addition, organotypic brain slice cultures from mouse or rat could be used to investigate the cellular and molecular processes of microglia *in vitro* (Stoppini et al., 1991; Gahwiler et al., 1997; Masuch et al., 2016). A final, more reductionistic approach would be to supply a 3D matrix with CNS microenvironmental cues or to coat the plastic or glass substrate with a surface that mimics the natural physicochemical properties that enhance microglial homeostasis.

## Concluding Remarks

The neuroscience field is increasingly appreciating that modulation of neuroinflammation is a promising strategy to beneficially affect the disease course of neurodegenerative diseases. Microglia are key players in neuroinflammatory responses, leading to an intensive research interest in this particular cell type. Many different microglia *in vitro* models have been established with respective advantages and limitations as summarized above. For high throughput screening assays, the microglial cell lines that are available might suffice, using culture conditions with or without serum. In addition, it is of importance that doubts on the species origin of human cell lines will be unequivocally confirmed and, if appropriate, accepted and corrected for. For research questions where it is important that the biology of M0 microglia from adult or elderly humans is reflected in the *in vitro* system, there is a paucity of good models. ESC and iPSC-derived models, in combination with exposure to CNS microenvironmental cues, form a good basis to establish models for M0 microglia. Such microglia will, however, not reflect the effects of aging. To model the latter, direct reprogramming of somatic cells might offer future possibilities. Alternatively, protocols have been established to isolate primary microglia from post-mortem CNS tissue of different species. Human primary microglia have been exposed to an aging (and/or diseased) CNS microenvironment and are not hampered by interspecies translation of the results. Primary microglia of other species are more readily available and can be obtained from post-mortem CNS tissue under more controlled conditions and with different transgenic backgrounds. The main challenge for primary microglia models is, however, to generate M0 microglia. Exposure of primary adult microglia to CNS-specific cues, combined with optimized cell culture protocols that avoid the use of serum, provide a good platform to *in vitro* model M0 microglia from adult or elderly humans.

The field is rapidly moving and we are gaining an ever better understanding of microglia biology. This will not only allow the development of better cell culture models, but will also enable the development of activation protocols that are more relevant for neurodegenerative disorders. The use of such refined approaches, will facilitate the development of innovative therapeutic approaches.

## Author Contributions

RT, SB, and JB designed, researched, and wrote the paper together.

## Conflict of Interest Statement

The authors declare that the research was conducted in the absence of any commercial or financial relationships that could be construed as a potential conflict of interest.
